# Colorectal cancer stage at diagnosis in male Florida firefighters

**DOI:** 10.1093/jncics/pkaf107

**Published:** 2025-11-05

**Authors:** Bonnie E Gould Rothberg, Tulay Koru-Sengul, Wei Zhao, Monique N Hernandez, Stephanie Negron, Victoria Pinilla, Natasha Schaefer Solle, Erin N Kobetz, Paulo S Pinheiro, David J Lee

**Affiliations:** Sylvester Comprehensive Cancer Center, University of Miami Miller School of Medicine, Miami, FL, United States; Sylvester Comprehensive Cancer Center, University of Miami Miller School of Medicine, Miami, FL, United States; Department of Public Health Sciences, University of Miami Miller School of Medicine, Miami, FL, United States; Sylvester Comprehensive Cancer Center, University of Miami Miller School of Medicine, Miami, FL, United States; Florida Cancer Data System, Sylvester Comprehensive Cancer Center, University of Miami Miller School of Medicine, Miami, FL, United States; Department of Public Health Sciences, University of Miami Miller School of Medicine, Miami, FL, United States; Department of Public Health Sciences, University of Miami Miller School of Medicine, Miami, FL, United States; Sylvester Comprehensive Cancer Center, University of Miami Miller School of Medicine, Miami, FL, United States; Department of Public Health Sciences, University of Miami Miller School of Medicine, Miami, FL, United States; Department of Medicine, University of Miami Miller School of Medicine, Miami, FL, United States; Sylvester Comprehensive Cancer Center, University of Miami Miller School of Medicine, Miami, FL, United States; Department of Public Health Sciences, University of Miami Miller School of Medicine, Miami, FL, United States; Department of Medicine, University of Miami Miller School of Medicine, Miami, FL, United States; Sylvester Comprehensive Cancer Center, University of Miami Miller School of Medicine, Miami, FL, United States; Department of Public Health Sciences, University of Miami Miller School of Medicine, Miami, FL, United States; Sylvester Comprehensive Cancer Center, University of Miami Miller School of Medicine, Miami, FL, United States; Department of Public Health Sciences, University of Miami Miller School of Medicine, Miami, FL, United States; Florida Cancer Data System, Sylvester Comprehensive Cancer Center, University of Miami Miller School of Medicine, Miami, FL, United States

## Abstract

**Background:**

Firefighters display increased risk of colorectal cancer (CRC). The distribution for CRC stage at diagnosis among firefighters compared to other occupational groups is unknown.

**Methods:**

Colorectal cancer cases from the Florida Cancer Data System cancer registry (2001-2014) for males ≥20 years were ascertained. Firefighters were identified through either direct linkage with Florida Fire Marshal’s Office employment and certification records or Standard Occupation Codes annotated at diagnosis. White-collar, blue-collar, and service workers were also identified according to these codes. Stage at diagnosis was classified as localized, regional, or distant. Bivariate tabulations of occupational groups and clinicodemographic covariates were conducted using contingency tables. The association between occupational group and stage at diagnosis was assessed by multivariable multinomial logistic regression adjusting for year of diagnosis, age, race and ethnicity, tobacco use and cancer diagnosis sequence. Adjusted odds ratios (aORs) are reported.

**Results:**

In total, 13 813 CRC cases, including 346 cases among firefighters, were analyzed. Firefighters were more likely to be non-Hispanic white and be diagnosed with a single primary cancer. One hundred and forty (40.5%) firefighter CRC cases were localized. Using firefighters as the referent group, only blue-collar workers approached 50% increased odds of later-stage diagnoses (aOR_Regional _= 1.44; 95% CI = 1.12 to 1.84; *P* = .004; aOR_Distant_ =1.49; 95% CI = 1.11 to 2.01; *P* = .008). Individuals ≥50 years and the first among multiple primary cancers were more likely to have localized CRC (*P* < .001).

**Conclusion:**

Although firefighters have increased CRC risk, they tend to be diagnosed more localized. Improved screening across worker groups can increase the percentage of localized CRCs.

## Graphical abstract

**Figure:**
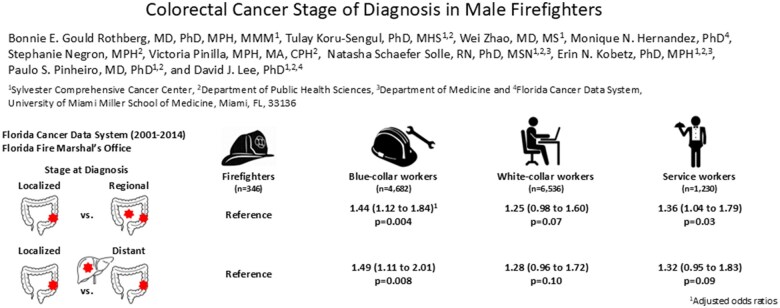

## Background

Occupational exposure as a firefighter is recognized as carcinogenic.^1^ Contributing exposures include substances from the fire effluent, flame retardants, firefighting foams, materials used in building construction, diesel exhaust fumes, and work-related lifestyle exposures such as shift-work and poor at-work dietary habits.^2^^,^^3^ Colorectal cancer (CRC) risk has been shown to be elevated by 33% in firefighters compared with population-based controls,^4^ with supporting data emerging as early as the mid-1980s.^5-7^ In 1 meta-analysis, a 33% increased mortality from rectal cancer has also been observed in this population.^8^

In the general population, environmental and lifestyle exposures such as obesity, increased consumption of red meat, lack of physical exercise, tobacco use, and alcohol consumption, are established CRC risk factors.^9^^,^^10^ Although direct evaluation of these exposures relative to CRC risk in firefighters is lacking, ingestion of polycyclic aromatic hydrocarbons (PAHs) from soot accumulated on clothes and equipment at the fire scene,^11^ as well as obesity rates exceeding 33% in the fire service,^12^ likely contribute. PAH-related genotoxicity and both obesity-related oxidative stress and chronic inflammation are established carcinogenic mechanisms and evidence for each of these effects has been demonstrated in firefighter biospecimens.^1^

Active screening for CRC can reduce morbidity and mortality.^13^^,^^14^ Screening guidelines were first formalized in 1980^15^ and the US Preventive Services Task Force (USPSTF) first recommended CRC screening in average-risk adults in 1996.^16^ Then, guidelines included annual fecal occult blood testing (FOBT) or sigmoidoscopy (unstated periodicity) beginning at 50 years in average-risk adults. Substantiative changes were made in 2008 where colonoscopy every 10 years was introduced as a screening option and the maximum age of recommended screening was capped at 75,^17^ and again in 2021, where the age to begin screening in the general population was lowered to 45 years.^18^

Stage at diagnosis impacts overall survival. In 2025, individuals with localized CRC that does not penetrate the visceral peritoneum (localized disease) will experience a 91% 5-year survival. By comparison, a 73% 5-year survival is observed for individuals with either local extension beyond the bowel wall or spread to pericolic, perirectal, paracolic, or inferior mesenteric lymph nodes (regional disease). Thirteen percent survival is observed for patients with metastases to other visceral organs (distant disease),^19^ rates that have largely remained constant since 2014.^20^

Several competing factors can influence stage at diagnosis. While adherence to screening recommendations can increase the number of cancers diagnosed at early stages, environmental exposures that trigger the development of aggressive tumors lead to CRCs that progress rapidly and present at an advanced stage in between screening intervals. Understanding patterns of stage at diagnosis can help discriminate populations with increased proportions of advanced CRC.

In this study, we utilize a curated database that links the registry of certified Florida firefighters with the Florida Cancer Data System (FCDS), the state cancer registry. We first characterize the distribution of CRC stage at diagnosis among firefighters (2001-2014) and then compare this distribution to those observed for other occupational groups as defined by the United States Bureau of Labor Statistics.

## Materials and methods

### Setting

This study uses incident cancer records from the FCDS as the analytical cohort. The FCDS was established in 1981 as the legislatively mandated, population-based cancer registry for Florida collecting all newly diagnosed cancer cases in the state. This study was approved by the Institutional Review Boards of the University of Miami and the Florida Department of Health. A waiver of informed consent was granted given that cancer data are a reportable event for purposes of cancer surveillance.

### Study population

All males in Florida diagnosed with CRC (ICD-O-3 codes C18.0, C18.2-18.9, C19.9, and C20.9) whose cancer was reported to the FCDS in 2001-2014 as either a single primary (sequence = 0) or the first or second case in individuals with multiple primary cancers (sequence = 1 or 2) and age at diagnosis ≥20 years were eligible. Exclusion criteria among firefighters were certification before the age of 20 and, for those identified through linkage with certification records, cancers diagnosed before the age of certification. Due to the small number of female firefighters diagnosed with CRC during 2001-2014 (*n* < 10), the study was limited to male firefighters and nonfirefighters.

### Occupation classification

Classification as a firefighter was primarily obtained through linkage of the FCDS database with firefighter employment and certification records from the Florida Fire Marshal’s Office (FMO) (1972-2012). Linkage methods were described previously.^21^ Briefly, the FMO has collected and maintained employment and certification records for all career and volunteer firefighters in Florida since 1972 when certification was mandated. Linkage-relevant variables provided by the FMO included name, address, date of birth, gender and, when available, social security number (SSN). These data were then submitted to LexisNexis for SSN validation and to obtain other missing identifiers. The linkage between the FMO and the FCDS datasets was performed using the FastLink software platform^22^ which applies the Fellegi-Sunter expectation-maximization algorithm for probabilistic modeling with allowances for missing data. Linkage was derived using SSN, home address, zip code, date of birth, and first and last names.

Firefighters not part of the FMO/FCDS linkage were identified from standardized information that the FCDS collects on case entries.^23^ Text indicating usual occupation (“type of job the patient engaged in for the greatest number of working years”) and usual industry (“type of business or industry where patient worked in his or her usual occupation”) (I/O codes) were referenced with individuals having US Bureau of Labor Statistics 2010 Standard Occupation Codes 3720, 3740, and 3750 classified as firefighters.^24^

Nonfirefighter occupations were derived from the same set of I/O codes. White-collar workers (eg, management, business, financial, professional, sales, and office occupations) mapped to I/O codes 0010-3540 and 4700-5940. Blue-collar workers (eg, construction, extraction, installation, repair, transportation) corresponded to I/O codes 6200-9000, 9030, 9040, 9050, and 9120-9750. Service jobs (eg, healthcare support, protective service, food preparation and serving, building and grounds cleaning and maintenance, and personal care) reflected codes 3600-4650, excluding codes 3720, 2740, and 3750, which were included as firefighters, as indicated above. Other occupations included codes 6010-6130, 9010, 9060, 9070, and 9100, which enumerated farm workers, homemakers, students, and disabled persons. Individuals missing an occupation code or coded by the FCDS as “unknown” (including individuals accessioned as “retired”) were excluded (*n* = 51 319).

### Stage at diagnosis

Tumor stage at diagnosis, classified as “localized,” “regional,” or “distant” using the Surveillance, Epidemiology and End-Results summary stage 2000 criteria,^25^ was the primary outcome. Individuals classified as “unstaged” were excluded.

### Covariates

Covariates included in multivariable models included year of diagnosis, age at diagnosis, race and ethnicity, tobacco use, and sequence of CRC diagnosis. Year at diagnosis was categorized as a 2-level variable stratified as 2001-2007 and 2008-2014, reflecting the timing for USPSTF CRC screening recommendation changes.^16-18^ Similarly, a 4-level variable was created with categories of 20-44 years (before recommended screening begins), 45-49 years (ages introduced in 2021 for general-risk population screening), 50-75 years (ages recommended for screening in the general population since 1996), and ≥76 years (age above which colonoscopy is no longer routinely recommended). A summary variable that combined race and ethnicity was created with categories of non-Hispanic White, non-Hispanic Black, and Hispanic; due to the small number of individuals collectively classified as either Native American, Asian, Asian Indian or Pakistani, or Pacific Islander (firefighters *n* < 10, total *n* < 350), these were excluded. Tobacco use was defined as current, former, never, and unknown. Sequence of CRC diagnosis distinguished single primaries (sequence = 0), first of multiple primaries (sequence = 1), and second of multiple primaries (sequence = 2).

### Data analysis

Frequency distributions were obtained for covariates across each occupational class and for both occupational group and covariates with stage at diagnosis. The association between occupational group (which excluded the heterogeneous “other” category [*n* = 1019]) and stage at diagnosis was assessed using multinomial logistic regression adjusting for year at diagnosis, age at diagnosis, race and ethnicity, tobacco use history, and sequence of CRC diagnosis. Adjusted odds ratios (aOR) and 95% confidence intervals for covariates are calculated where localized stage at diagnosis was chosen as the referent group. Given the 28 unique multinomial logistic regression comparisons, statistical significance was a priori set at *P* = .002 (0.05/28). Data management and statistical analyses were performed in SAS version 9.4 (SAS Institute, Cary, NC). Power calculations were executed using PASS 2024 v24.0.2 to estimate the minimum detectable aOR to reach at least 80% power for a multivariate logistical regression model separately for regional versus localized disease and distant versus localized disease across occupational groups (NCSS, LLC, Kaysville, UT).

## Results

A total of 13 813 cases of CRC with a coded occupational group were identified in the FCDS from 2001 to 2014, of which 12 794 were classified as either firefighters (*n* = 346; 2.7%), white-collar (*n* = 6536; 51.1%), blue-collar (4682; 36.6%), or service workers (1230; 9.6%). Compared with other occupation groups, firefighters were more likely to be non-Hispanic White and have their CRC diagnosed as a single primary cancer (Table 1). Two hundred and seventy-two firefighters (78.6%) were identified through linkage with the FMO and the remaining 74 (21.4%) were identified according to I/O codes.

**Table 1.** Characteristics of male participants diagnosed with colorectal cancer by occupation group, 2001-2014.

Covariate	Total sample (*n* = 13 813)	Firefighters (*n* = 346)	White-collar workers (*n* = 6536)	Blue-collar workers (*n* = 4682)	Service workers (*n* = 1230)	Other workers^a^ (*n* = 1019)
Year of diagnosis						
2001-2007	7078 (51.2%)^b^	178 (51.4%)	3484 (53.3%)	2374 (50.7%)	564 (45.9%)	478 (46.9%)
2008-2014	6735 (48.8%)	168 (48.6%)	3052 (46.7%)	2308 (49.3%)	666 (54.1%)	541 (53.1%)
Age at diagnosis, years						
20-44	923 (6.7%)	26 (7.5%)	393 (6.0%)	279 (6.0%)	116 (9.4%)	109 (10.7%)
45-49	935 (6.8%)	31 (9.0%)	375 (5.7%)	351 (7.5%)	96 (7.8%)	82 (8.0%)
50-75	9178 (66.4%)	228 (65.9%)	4189 (64.1%)	3184 (68.0%)	819 (66.6%)	758 (74.4%)
76+	2777 (20.1%)	61 (17.6%)	1579 (24.2%)	868 (18.5%)	199 (16.2%)	70 (6.9%)
Race and ethnicity						
Non-Hispanic White	10 982 (79.5%)	315 (91.0%)	5626 (86.1%)	3577 (76.4%)	874 (71.1%)	590 (57.9%)
Non-Hispanic Black	1428 (10.3%)	16 (4.6%)	375 (5.7%)	600 (12.8%)	205 (16.7%)	232 (22.8%)
Hispanic	1403 (10.2%)	15 (4.3%)	535 (10.8%)	505 (10.8%)	151 (12.3%)	197 (19.3%)
Tobacco use						
Never	5009 (36.3%)	138 (39.9%)	2652 (40.6%)	1402 (29.9%)	429 (34.9%)	388 (38.1%)
Former	3971 (28.7%)	79 (22.8%)	1905 (29.1%)	1444 (30.8%)	343 (27.9%)	200 (19.8%)
Current	1958 (14.2%)	45 (13.0%)	673 (10.3%)	847 (18.1%)	188 (15.3%)	205 (20.1%)
Unknown	2875 (20.8%)	84 (24.3%)	1306 (20.0%)	989 (21.1%)	270 (22.0%)	226 (22.2%)
Sequence of CRC diagnosis						
Single primary cancer	8250 (59.7%)	264 (76.3%)	3850 (58.9%)	2788 (59.5%)	699 (56.8%)	649 (63.7%)
First of multiple primary cancers	2617 (18.9%)	55 (15.9%)	1237 (18.9%)	904 (19.3%)	249 (20.2%)	172 (16.9%)
Second of multiple primary cancers	2946 (21.3%)	27 (7.8%)	1449 (22.2%)	990 (21.1%)	282 (22.9%)	198 (19.4%)

a Other occupations include farm workers, homemakers, disabled individuals, and students.

b Percentages may not sum to 100% due to rounding.

Overall, firefighters (*n* = 140 of 346 firefighters; 40.5%) were the most likely to have their CRC diagnosed at a localized stage whereas blue-collar workers displayed the highest proportions of regional (2018 of 4682; 43.1%) and distant stage (1155 of 4682; 24.7%) at diagnosis. Individuals over the age of 50 (4002 of 11 127; 36.0%) and non-Hispanic White individuals (3696 of 12 794; 35.6%) displayed the highest proportions of localized CRCs compared with other age or race and ethnicity groups, respectively. Current smokers displayed higher proportions of regional (766 of 1753; 43.7%) or distant (443 of 1753; 25.3%) CRC compared with never or former smokers; 39.3% (962 of 2445) of individuals with the first of multiple primary cancers were diagnosed with localized disease whereas only 32.4% of individuals with a single primary cancer or 37.0% of individuals having CRC as their second of multiple primary cancers presented with localized disease. Compared with cancers from 2001 to 2007 (1427 of 6600 [21.6%]), those diagnosed during 2008-2014 had an excess of distant stage diagnoses (1564 of 6194 [25.3%]) (Table 2).

**Table 2.** Characteristics of Florida males with annotated occupation group diagnosed with colorectal cancer by SEER (Surveillance, Epidemiology, and End-Results) stage, 2001-2014.

Covariate		SEER stage at diagnosis		
	Total individuals No. (Col %)^a^	Localized stage; No. (Row %)	Regional stage; No. (Row %)	Distant stage; No. (Row %)
All (Row %)	12 794^b^	4442 (34.7%)	5361 (41.9%)	2991 (23.4%)
Occupation group				
Firefighters	346 (2.7%)	140 (40.5%)	133 (38.4%)	73 (21.1%)
White collar	6536 (51.1%)	2382 (36.4%)	2679 (41.0%)	1475 (22.6%)
Blue collar	4682 (36.6%)	1509 (32.2%)	2018 (43.1%)	1155 (24.7%)
Service	1230 (9.6%)	411 (33.4%)	531 (43.2%)	288 (23.4%)
Year of diagnosis				
2001-2007	6600 (51.6%)	2388 (36.2%)	2785 (42.2%)	1427 (21.6%)
2008-2014	6194 (48.4%)	2054 (33.2%)	2576 (41.6%)	1564 (25.3%)
Age at diagnosis, years				
20-44	814 (6.4%)	203 (24.9%)	383 (47.1%)	228 (28.0%)
45-49	853 (6.7%)	237 (27.8%)	379 (44.4%)	237 (27.8%)
50-75	8420 (65.8%)	2866 (34.0%)	3521 (41.8%)	2033 (24.1%)
76+	2707 (21.2%)	1136 (42.0%)	1078 (39.8%)	493 (18.2%)
Race and ethnicity				
Non-Hispanic White	10 392 (81.2%)	3696 (35.6%)	4324 (41.6%)	2372 (22.8%)
Non-Hispanic Black	1196 (9.3%)	355 (29.7%)	524 (43.8%)	317 (26.5%)
Hispanic	1206 (9.4%)	391 (32.4%)	513 (42.5%)	302 (25.0%)
Tobacco use				
Never	4621 (36.1%)	1602 (34.7%)	1947 (42.1%)	1072 (23.2%)
Former	3771 (29.5%)	1340 (35.5%)	1582 (42.0%)	849 (22.5%)
Current	1753 (13.7%)	544 (31.0%)	766 (43.7%)	443 (25.3%)
Unknown	2649 (20.7%)	956 (36.1%)	1066 (40.2%)	627 (23.7%)
Sequence of CRC diagnosis				
Single primary cancer	7601 (59.4%)	2464 (32.4%)	3241 (42.6%)	1896 (24.9%)
First of multiple primary cancers	2445 (19.1%)	962 (39.3%)	1055 (43.1%)	428 (17.5%)
Second of multiple primary cancers	2748 (21.5%)	1016 (37.0%)	1065 (38.8%)	667 (24.3%)

a Percentages may not sum to 100% due to rounding.

b Excludes individuals whose occupations were classified as “other.”

Multivariable multinomial logistic regression comparing the odds of stage at diagnosis using localized stage as the referent group while adjusting for occupational group, year of diagnosis, age at diagnosis, race and ethnicity, tobacco use, and sequence of CRC diagnosis was fitted (Table 3). Following Bonferroni adjustment, individuals between the ages of 50-75 years or ≥76 years were less likely to be diagnosed as regional (aOR_50-75_ = 0.67; 95% CI = 0.56 to 0.80; *P* < .001 and aOR_≥76_ = 0.54; 95% CI = 0.44 to 0.65; *P* < .001) or distant disease (aOR_50-75 _= 0.66; 95% CI = 0.54 to 0.80; *P* < 0.001 and aOR_≥76 _= 0.41; 95% CI = 0.33 to 0.51; *P* < .001) compared with referent 20-44 year olds. We also noted significant associations for sequence of CRC diagnosis where, compared to individuals with a single cancer diagnosis, individuals where their CRC was their first of multiple primary cancers were less likely to be diagnosed at a regional (aOR = 0.84; 95% CI = 0.76 to 0.93; *P* < .001) or distant stage (aOR = 0.59; 95% CI = 0.52 to 0.67; *P* < .001).

**Table 3.** Multivariable multinomial logistic regression of occupational, demographic, and clinical covariates across SEER (Surveillance, Epidemiology, and End-Results) stage at diagnosis (total eligible *n* = 12 794).^a^

Covariate	Regional versus localized ^a^OR (95% CI)	*P* ^b^	Distant versus localized ^a^OR (95% CI)	*P*
Occupational group				
Firefighters	Ref.		Ref.	
White collar	1.25 (0.98 to 1.60)	.07	1.28 (0.96 to 1.72)	.10
Blue collar	1.44 (1.12 to 1.84)	.004	1.49 (1.11 to 2.01)	.008
Service	1.36 (1.04 to 1.79)	.03	1.32 (0.95 to 1.83)	.09
Year of diagnosis				
2001-2007	Ref.		Ref.	
2008-2014	1.07 (0.99 to 1.16)	.10	1.24 (1.13 to 1.36)	<.001
Age at diagnosis, years				
20-44	Ref.		Ref.	
45-49	0.84 (0.67 to 1.07)	.16	0.89 (0.68 to 1.15)	.36
50-75	0.67 (0.56 to 0.80)	<.001	0.66 (0.54 to 0.80)	<.001
76+	0.54 (0.44 to 0.65)	<.001	0.41 (0.33 to 0.51)	<.001
Race and ethnicity				
Non-Hispanic White	Ref.		Ref.	
Non-Hispanic Black	1.16 (1.00 to 1.34)	.05	1.23 (1.05 to 1.45)	.01
Hispanic	1.04 (0.90 to 1.20)	.59	1.07 (0.91 to 1.26)	.39
Tobacco use				
Never	Ref.		Ref.	
Former	1.03 (0.93 to 1.14)	.60	1.03 (0.92 to 1.16)	.60
Current	1.10 (0.96 to 1.25)	.17	1.14 (0.98 to 1.33)	.09
Unknown	0.92 (0.83 to 1.03)	.14	0.98 (0.87 to 1.12)	.80
Sequence of CRC diagnosis				
Single primary cancer	Ref.		Ref.	
First of multiple primary cancers	0.84 (0.76 to 0.93)	<.001	0.59 (0.52 to 0.67)	<.001
Second of multiple primary cancers	0.84 (0.75 to 0.93)	<.001	0.92 (0.82 to 1.04)	.18

a Adjusted for occupational group, year of diagnosis, age at diagnosis, race and ethnicity, tobacco use, and sequence of CRC diagnosis.

b *P* values ≤.002 are significant.

Using firefighters as the referent group, after Bonferroni adjustment, no significant associations with stage were observed with occupational categorization. Although blue-collar workers demonstrated nearly 50% increased odds of later-stage diagnoses (aOR_Regional_ = 1.44; 95% CI = 1.12 to 1.84; *P* = .004; aOR_Distant_ = 1.49; 95% CI = 1.11 to 2.01; *P* = .008), the comparatively small number of firefighters may have contributed to these values not achieving statistical significance. There were no significant differences between firefighters and service workers, the group that they derive from, with respect to stage at diagnosis (*P* > .01) (Table 3).

## Discussion

Stage at diagnosis is a significant prognostic factor for CRC mortality. In this manuscript, we compared the distribution of stage at diagnosis for CRC between firefighters and standard occupational groupings of white-collar, blue-collar, and service industries while adjusting for demographic and lifestyle covariates. Overall, 40.5% of CRCs in firefighters were diagnosed as localized tumors, a value qualitatively higher than the remaining occupational groups. Career firefighters typically receive insurance directly through their employer or through the union. This facilitates access to screening, possibly contributing to the overall high rates of localized stage at diagnosis among firefighters. Nonetheless, firefighters did not show a significant difference in stage at diagnosis from other service workers of which they form a subset or from white-collar workers on multivariable analysis.

In contrast, blue-collar workers displayed nearly 50% increased odds of regional or distant stage compared with firefighters, though this was not statistically significant. Previous research has shown that, compared to other occupational groups, blue-collar workers are less likely to complete preventive health interventions, including CRC screening. One study demonstrated that inflexible work schedules and lack of sick days prevented blue-collar workers from attending regular doctor visits.^26^ Taking multiple days off to allow for a bowel preparation could be prohibitive. Blue-collar workers have lower socioeconomic status, lower health literacy and are underinsured compared with other workers. In one study, blue-collar workers were up to 30% less likely to have undergone CRC screening, an association that was eliminated following adjustment for socioeconomic and insurance statuses.^27^ Taken together, these factors can explain the observed differences in stage at diagnosis between firefighters and blue-collar workers.

Additional competing factors contribute to determining stage at diagnosis. Length-time bias stipulates that localized tumors, particularly those diagnosed during routine screening, are more likely to be indolent whereas aggressive tumors may develop and rapidly grow in between screening intervals to subsequently present with distant metastases.^28^ Colonoscopies can also themselves serve as a therapeutic tool in that, through the removal of precancerous polyps, potential lesions are no longer at risk for progressing beyond the premalignant state. In one landmark study, after the removal of colorectal adenomas, zero symptomatic CRC cases evolved over 6 years of follow-up.^29^ A second analysis from the same parent study showed a 53% reduction in CRC mortality following 15.8 years of follow-up.^30^ Our exclusion of in situ lesions has prevented us from directly measuring this benefit.

Data have further shown correlations between specific carcinogens and development of distinct molecular phenotypes. For example, a large population-based case-control study demonstrated the association between consumption of a western diet and the incidence of p53 mutations, an adverse CRC prognostic factor.^31^ Similarly, a meta-analysis revealed a positive association between body mass index and the adversely prognostic microsatellite-stable CRC phenotype.^32^ Consequently, despite adherence to a screening regimen, individuals who consume western diets or who are obese may nonetheless have increased risk for developing cancers that present at advanced stage between screening intervals.

Screening participation could also explain the significant differences in distribution of stage at diagnosis between individuals over and under the age of 50, the threshold for which recommended screening began during our study interval.^15^ Additionally, the slightly elevated risk for late-stage malignancies among 50- to 75-year-olds compared with individuals older than 75 years could be due to the excess diagnosis of advanced cancers at the time of an individual’s first screening.

Compared with individuals diagnosed with a single primary cancer or with the second of multiple primary cancers, CRCs occurring as the first of multiple primary cancers displayed the most favorable staging distribution. As individuals for whom their CRC is not their final cancer diagnosis must survive long enough to develop a subsequent de novo cancer, this favors the likelihood for their CRC to be localized.

Finally, we observed a higher percentage of late-stage cancers in the period between 2008 and 2014 compared with the period between 2001 and 2007. Altogether, cases annotated as “unstaged” are frequently missing the “M” (distant metastasis: yes/no) component of the staging rubric.^33^ Moreover, an excess of “unstaged” cancers, especially in earlier calendar years, represent cases with advanced stage.^34^^,^^35^ Consequently, our distribution toward a higher percentage of distantly staged cancers during the period between 2008 and 2014 likely reflects increased precision regarding staging during this period.

Strengths of our study include the use of a population-based cancer registry, linkage with firefighter employment and certification records to accrue a large cohort of firefighters, and the inclusion of a 14-year timespan during which CRC screening guidelines, including the eligible age range as well as the use of FOBT and sigmoidoscopy, were largely stable. Despite the introduction of colonoscopy to formal screening recommendations in 2008, this did not yield an excess of CRC diagnoses in the second half of our time interval.

At the same time, several limitations are recognized. First, due to the relatively small number of firefighters included in the study (*n* = 346), our power was limited. For example, for the relationship between firefighters and white-collar workers, the minimum detectable aOR to achieve 80% power for the regional versus localized disease comparison was 1.42 and for distant versus localized disease was 1.52. Similarly, the minimal detectable aOR between service workers and firefighters for distant versus localized disease was 1.52. Each is higher than our reported aORs.

Next, due to the small number (<10) of female firefighters, we limited our study to males. Female firefighters altogether only comprise 7%-9% of the firefighter workforce.^36^ Accruing data from more recent years could increase representation of female firefighters, allowing for a meaningful analysis. Expanding our linkage between the FCDS and the Florida FMO is ongoing.

Third, annotation of usual occupation for nonfirefighters in the FCDS is incomplete. Of the 65 132 individuals identified with an eligible CRC, only 19.6% had an occupation classified as either firefighter, white-collar worker, blue-collar worker or service worker, with the remaining 80.4% annotated as “other occupation” or “unknown/missing occupation.” The level of I/O code missingness measured during our study period has been well-documented among other cancer registries. The New Hampshire State Cancer Registry documented 74.5% missing data for the 2005 diagnostic year^37^ and the California Cancer Registry similarly noted over 60% with blank, uncodable, or vague annotations such as “retired.”^38^ Missingness is also likely not at random. Query of I/O codes in the FCDS identified 74 firefighters with CRC not linked through the FMO database, likely representing firefighters certified out of state.^39^ However, we only identified an additional 1230 service workers, suggesting a high rate of missingness among nonfirefighter service workers. Our bias toward more complete identification of firefighters compared with other employment categories risks misclassifying associations toward the null even if a true distinction in stage at diagnosis was present.

Finally, we report on an FCDS data subset collected between 2001 and 2014, which may limit conclusions regarding current distribution of stage at diagnosis. Nonetheless, recent aggregate data reflecting stage of CRC diagnosis show only a minor decrease in the proportion of cases diagnosed at a distant stage among individuals aged 50-75 from 23% in 2015 to 20% in 2022,^40^ suggesting that our data are consistent with current trends.

In conclusion, our findings indicate that firefighters have a similar distribution of CRC stage at diagnosis compared to other service workers but may have increased odds of localized disease relative to blue-collar workers. This trend suggests that firefighters may engage more actively in screening programs, potentially facilitating earlier detection. However, with over 60% of diagnoses occurring at regional or distant stages, these results also underscore the potential role for occupation-related exposures to drive more aggressive disease presentations between screening intervals. Together, these insights highlight the importance regarding how occupational hazard mitigation such as encouraging decontamination of equipment and doffing turnout gear prior to reentering the fire station, thorough laundering of turnout gear using commercial extractors, providing healthy nutritional options at the fire station and educating firefighters regarding how to pursue these at home, as well as enhanced screening efforts, may shift CRC diagnosis among firefighters toward earlier, more treatable stages.

## Data Availability

The datasets presented in this article are not readily available because they are governed by strict confidentiality agreements between the University of Miami and the Florida Department of Health, the Florida Cancer Data System, and the Florida Fire Marshal’s Office. Requests to access the datasets should be directed to the Florida Department of Health: health@flhealth.gov.
